# Long-term fate of etoposide-induced micronuclei and micronucleated cells in Hela-H2B-GFP cells

**DOI:** 10.1007/s00204-020-02840-0

**Published:** 2020-07-17

**Authors:** Hauke Reimann, Helga Stopper, Henning Hintzsche

**Affiliations:** 1 Institute of Pharmacology and Toxicology, University of Würzburg, Versbacher Straße 9, 97078 Würzburg, Germany; 2 Bavarian Health and Food Safety Authority, Eggenreuther Weg 43, 91058 Erlangen, Germany

**Keywords:** Micronuclei, Cell fate, Etoposide, Live imaging, DNA damage

## Abstract

Micronuclei are small nuclear cellular structures containing whole chromosomes or chromosomal fragments. While there is a lot of information available about the origin and formation of micronuclei, less is known about the fate of micronuclei and micronucleated cells. Possible fates include extrusion, degradation, reincorporation and persistence. Live cell imaging was performed to quantitatively analyse the fates of micronuclei and micronucleated cells occurring in vitro. Imaging was conducted for up to 96 h in HeLa-H2B-GFP cells treated with 0.5, 1 and 2 µg/ml etoposide. While a minority of micronuclei was reincorporated into the main nucleus during mitosis, the majority of micronuclei persisted without any alterations. Degradation and extrusion were observed rarely or never. The presence of micronuclei affected the proliferation of the daughter cells and also had an influence on cell death rates. Mitotic errors were found to be clearly increased in micronucleus-containing cells. The results show that micronuclei and micronucleated cells can, although delayed in cell cycle, sustain for multiple divisions.

**Electronic supplementary material:**

The online version of this article (10.1007/s00204-020-02840-0) contains supplementary material, which is available to authorized users.

## Introduction

Micronuclei are small nuclear cellular structures usually formed during mitosis. They can contain whole chromosomes or chromosomal fragments, which can be distinguished by the presence of kinetochores in the micronuclei (Norppa and Falck 2003). Micronuclei are used as end point for chromosomal mutation in the micronucleus test, which is routinely conducted in genotoxicity testing (OECD 2016). Furthermore, the frequency of micronuclei in peripheral lymphocytes is considered predictive for the cancer risk in humans (Bonassi et al. 2007). Typical causes for the formation of micronuclei are, amongst others, spindle attachment defects or DNA double-strand breaks (Kisurina-Evgenieva et al. 2016). While there is a lot of information available about the origin and formation of micronuclei, less is known about the fate of the micronuclei and micronucleated cells. Only a limited amount of systematic studies have addressed the fate of micronuclei, but some possibilities have been described during the last years, e.g. extrusion, degradation, reincorporation and persistence of micronuclei (Hintzsche et al. 2017; Stopper and Hintzsche 2019). In addition, micronuclei are discussed to be involved in chromothripsis, which could explain the local occurrence of massive chromosomal damages (Crasta et al. 2012; Luijten et al. 2018). Thus, micronuclei could cause additional chromosomal instability and therefore contribute to cell transformation and carcinogenesis, instead of only being a biomarker (Ye et al. 2019).

Furthermore, it is not clear if and to what extent DNA in micronuclei is replicated, transcribed or repaired (Hintzsche et al. 2017). Crucial for correct function of these processes in micronuclei is the integrity of the micronuclear envelope. Envelope defects cause an impaired import and export of proteins into the micronucleus and have been shown to reduce DNA damage repair, which in turn could lead to chromothripsis (Terradas et al. 2016). One mechanistic explanation for this might be incorrect lamin B1 assembly which leads to micronucleus disruption (Hatch et al. 2013). Breakdown of the micronucleus, e.g. during chromothripsis, could lead to increased accumulation of cyclic GMP-AMP synthase, inducing a proinflammatory response to micronuclear chromatin via interferon-stimulated gene expression (Mackenzie et al. 2017). In addition, proteasome function and chromatin compaction are abnormal in micronuclei compared to the main nuclei (Maass et al. 2018).

Live cell imaging allows the observation of dynamic processes for a certain amount of time and is a powerful technique to analyse the spatiotemporal distribution of cellular components. A live cell imaging setup consists of a fluorescence microscope (widefield or confocal) and a chamber to maintain an optimal environment for living cells for hours to days (Ettinger and Wittmann 2014). Live cell imaging has previously been used for the investigation of micronuclei. For example, four novel mechanisms of formation of micronuclei were postulated in a live cell imaging study, in addition to three already known mechanisms (Huang et al. 2011a). Another study using HeLa cells stably transfected with H2B-GFP investigated the cellular fate after treatment with hydroxyurea (Utani et al. 2010). However, all previous studies have a number of limitations, particularly investigating only one out of a number of putative fates and/or following cells only for a short-term duration covering mostly less than one cell cycle period.

For a successful live cell imaging experiment, three main factors should be considered: phototoxicity, signal-to-noise-ratio and spatiotemporal resolution (Laissue et al. 2017). Among these factors, phototoxicity is crucial in particular for long-term experiments, as it may influence the results and invalidate the findings. Nevertheless, there is no general standard test to evaluate phototoxicity, which makes it necessary to find an individual way to address it and to closely examine cells for any signs of toxic effects caused by the fluorescence (Laissue et al. 2017). Ways to reduce phototoxicity are using narrow-band filters, lower light intensity instead of shorter exposure time or the use of fluorophores with excitation at longer wavelengths that need lower-energy light for activation (Icha et al. 2017; Magidson and Khodjakov 2013). Signal-to-noise ratio as well as spatiotemporal resolution is important especially in micronuclei research, as the size of a micronucleus is comparatively small and certain events could be overlooked easily. The maximum possible signal-to-noise ratio and spatiotemporal resolution are furthermore dependent on the properties of the available live cell imaging system.

We conducted long-term live cell imaging of micronuclei and micronucleated cells for up to 96 h to get a deeper insight into the long-term consequences of micronucleus formation. HeLa-H2B-GFP cells allowed us to identify and track nuclei and micronuclei. Cells were treated with etoposide, which is a chemotherapeutic known to induce high frequencies of micronuclei and is therefore an appropriate model substance for a study on the investigation of micronuclei and micronucleated cells via live cell imaging (Fowler et al. 2010).

## Material and methods

### Cell culture and treatment conditions

For all experiments, Hela cells stably transfected with H2B-GFP were used (provided by Noriaki Shimizu, Graduate School of Integrated Sciences for Life, Hiroshima University, Japan) (Kanda et al. 1998; Utani et al. 2010). Cells were cultivated at 37 °C and 5% CO_2_ in DMEM High Glucose (Sigma-Aldrich) without phenol red, but supplemented with 10% FCS (Merck), 2 mM l-glutamine (Sigma), 100 µg/ml streptomycin (Sigma), 100 U/ml penicillin (Sigma), 1 mM sodium pyruvate (Sigma) and 25 mM HEPES (Sigma). Cells were tested regularly for mycoplasms with Hoechst 33,342 staining and used up to 40 passages after thawing. Etoposide was purchased from Teva, and cells were treated for 3 h at concentrations of 0.5, 1 and 2 µg/ml. The solvent was DMSO.

### Cytokinesis-block micronucleus test

To preliminarily confirm that the doses used induce micronuclei without relevant cytotoxicity, a cytokinesis-block micronucleus test was performed with fixed cells. After treatment and medium change, 1.5 µg/ml cytochalasin B was added for 24 h and micronuclei in binuclear cells were analysed as described before (Fenech 2007). Mono-, bi- and multinuclear as well as mitotic and apoptotic cells were evaluated in 1000 cells, and micronuclei were scored in 1000 binucleated cells. Each analysis was performed on two slides.

### Time-lapse imaging

After treatment of cells, the medium was changed and cells were cultivated until the next day to allow for micronucleus formation. On the next day, cells were placed in the plate chamber of a Nikon Ti–S fluorescence microscope equipped with a motorized table and an incubation envelope (Okolab) to maintain temperature, CO_2_ and humidity. The system was controlled by the software NIS-Elements Advanced Research version 5.10.01 (Nikon). The positions of the chosen micronucleated cells were saved with the software. Images were taken every 10 min for 96 h, covering up to four to five mitoses (cell cycle durations). The originally selected cells are denominated F0, whereas the following generations are denominated F1–F5. Subsequently, sequences for each position were generated. A 40 × 0.75 NA objective (Nikon) and an Andor Luca S (Andor Technology) camera with ND 64 and exposure time of 100 ms were used without binning. Each experiment was repeated five times. Two types of untreated controls were included, one subtype was micronucleated cells and the other subtype was micronucleus-free cells.

### Evaluation

The occurrence and timing of mitosis, cell death or cell arrest was noted. Cell death was defined as shrinking of the nucleus along with increased signal intensity or swelling of the nucleus with reduced signal intensity. These findings may indicate apoptosis or necrosis, but a clear differentiation was not possible based on morphological criteria.

The duration of mitosis (from the detection of prophase to telophase) and interphase as well as occurrence of mitotic errors such as fusion of two nuclei before mitosis or mitosis to more than two nuclei/fragments after mitosis was evaluated. Cells, which could not be followed further, e.g. because they left the field of view, were excluded from the final data analysis. The presence of micronuclei for each cell and generation was also noted. Events like extrusion, degradation or if micronuclei could be followed during next mitosis were counted. Micronuclei were counted for each generation individually, as usually no direct tracking of micronuclei during mitosis was possible. Persistence was assumed, when the number of micronuclei after mitosis was equal to the number before mitosis, whereas reincorporation was assumed, when the micronucleus number after mitosis was smaller than before. More micronuclei after mitosis were considered as new formation. 30 micronucleated cells and their respective daughter cells were analysed per treatment and experiment except for the micronucleus-free control subgroup in which only ten non-micronucleated cells and respective daughter cells were tracked. All experiments were performed five times.

## Results

To determine appropriate low, medium and high doses of etoposide, a preliminary micronucleus test was performed to find doses inducing micronuclei without relevant cytotoxicity. 0.5, 1 and 2 µg/ml etoposide were found to be doses fulfilling these criteria and were therefore used for the following experiments (Supplementary Fig. 1). The live imaging experiments covered 96 h, which allows to track the fate of micronuclei and micronucleated cells for multiple cell cycles (Supplementary Figs. 2, 3).

### Fate of micronuclei

**Fig. 1:**
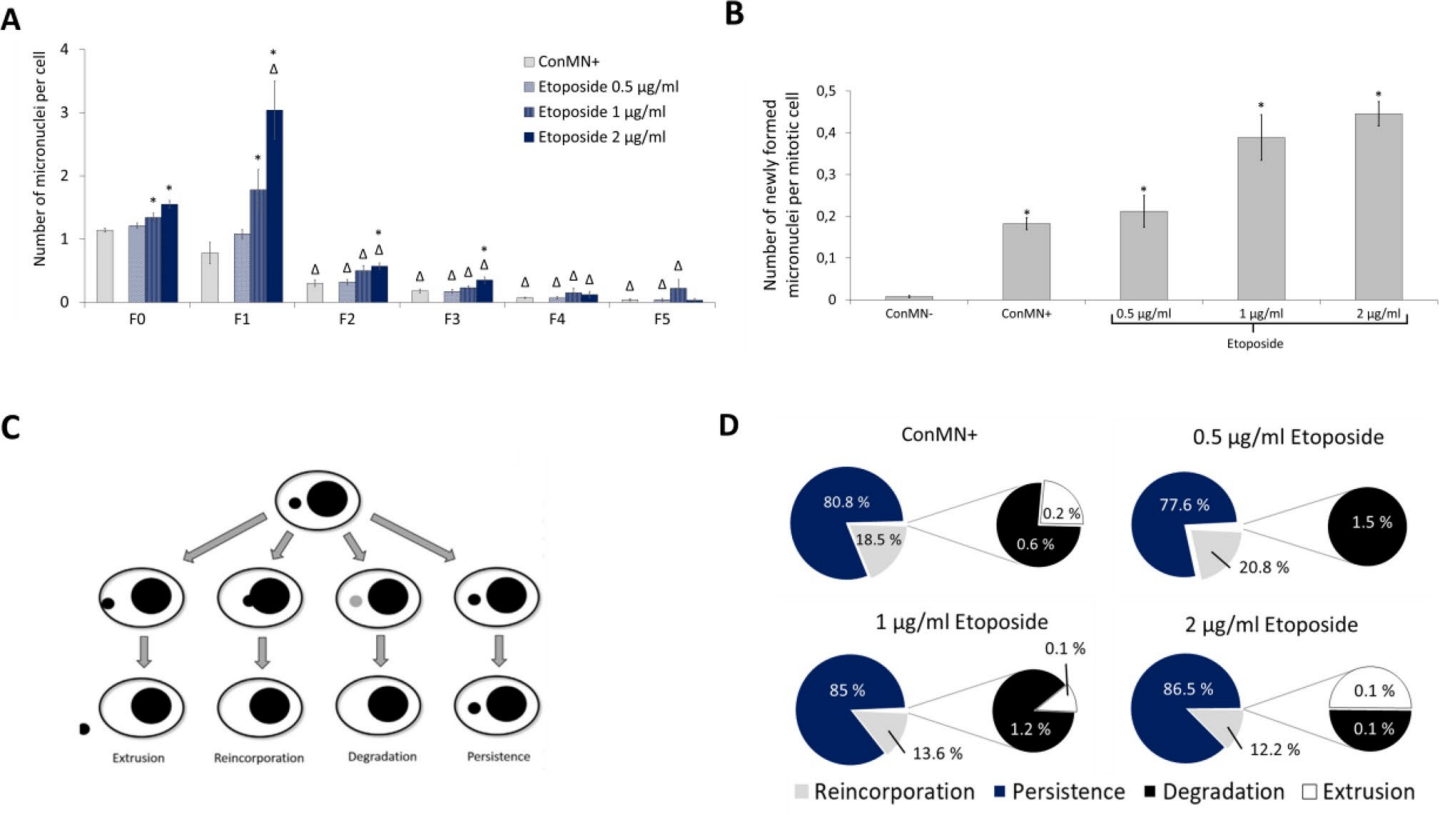
Fate of micronuclei. **a** Micronuclei per cell in each generation. All presented values are mean out of five experiments with standard error. Asterisk represents *p* < 0.05 compared to micronucleated control cells (ConMN +); delta represents *p* < 0.05 to value of the treatment in F0 (*t* test). **b** Newly formed micronuclei in controls (non-micronucleated control cells (ConMN −)/ConMN +) and after treatment with etoposide. Number of micronuclei per mitosis in each generation. All presented values are mean out of five experiments with standard error. Asterisk represents *p* < 0.05 compared to ConMN − (*t* test). **c** Possible fates of micronuclei: extrusion, reincorporation, degradation and persistence. Based on Hintzsche et al. 2017. **d** Observed fates of micronuclei within a cell cycle averaged over all generations: extrusion, reincorporation, degradation and persistence

When we observed micronucleus-containing cells in time-lapse imaging, we found an increase in the number of micronuclei per cell at F0 and F1 in all treatment groups, which means that some cells contained more than one micronucleus, and a decrease over time in the following generations, meaning that some cells had lost or reintegrated the micronuclei or formed daughter cells without micronuclei (Fig. 1a). In F0 and F1 and to a lower extent also in F2 and F3, a dose-dependent increase in the number of micronuclei per cell was observed. No evaluation of micronuclei that might have formed in non-micronucleated control cells during the observation period was conducted, as the number of micronuclei, which could only be introduced via new formation in daughter cells, was too low for analysis. There was a significant dose-dependent increase of newly formed micronuclei per mitosis in micronucleated cells with and without etoposide treatment, when compared to non-micronucleated control cells (Fig. 1b).

Extrusion, reincorporation, degradation and persistence are possible fates of micronuclei (Fig. 1c, d). The majority of micronuclei, which were observed with live cell microscopy, persisted during the following cell cycle including mitosis. Reincorporation occurred in 10–20% of all observed micronuclei (Fig. 1c, d). Degradation and extrusion were observed only rarely or never, while the fate of around 10% of all micronuclei could not be determined reliably and these were therefore excluded from analysis.

### Proliferation and cell death in micronucleated and non-micronucleated cells

**Fig. 2:**
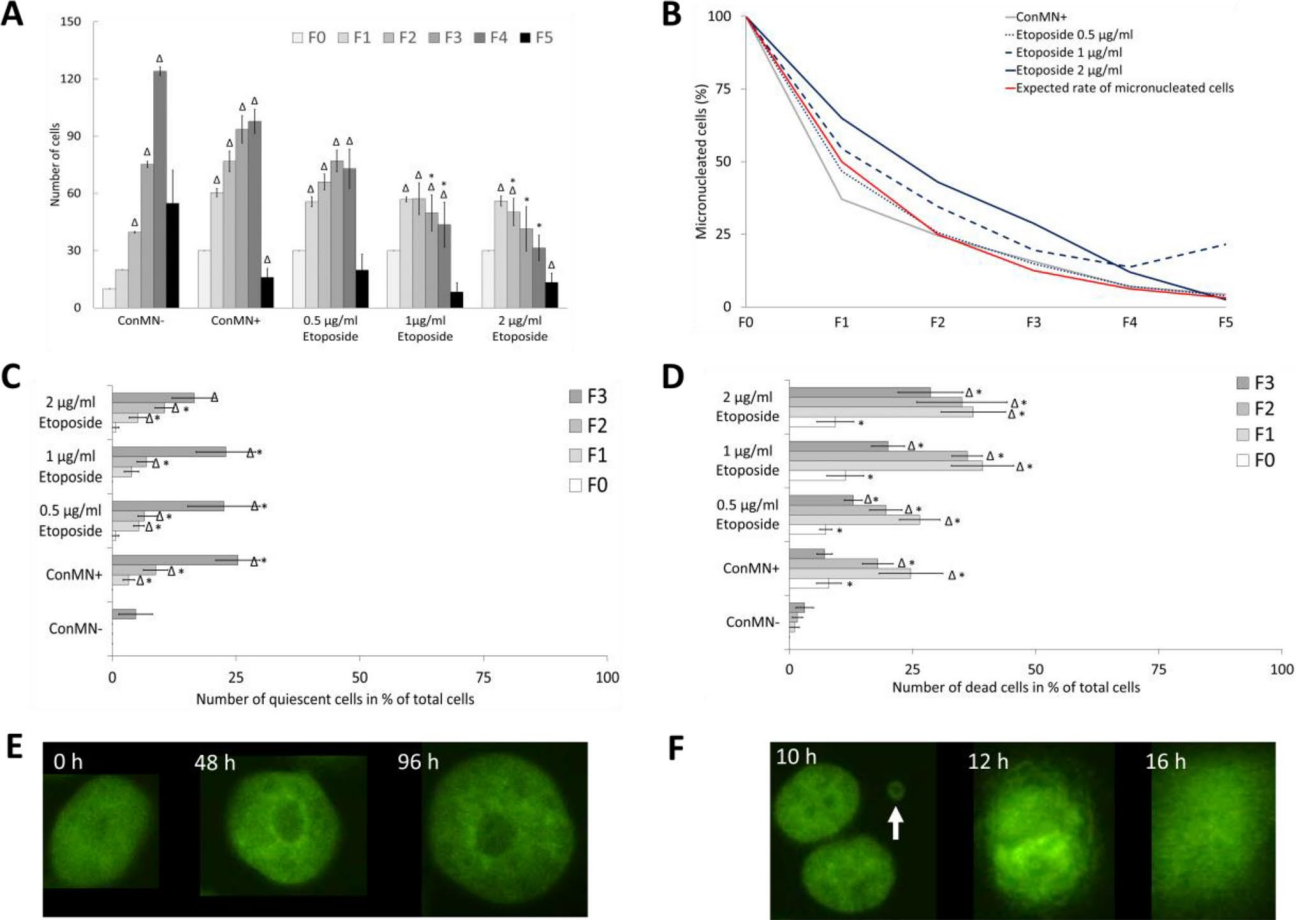
Cell number, cell death and arrest. **a** Number of cells after treatment with etoposide and controls (non-micronucleated control cells (ConMN−)/micronucleated control cells (ConMN +)) in generations F0–F5. All presented values are mean out of five experiments with standard error. Asterisk represents *p* < 0.05 compared to ConMN + ; delta represents *p* < 0.05 to value of the treatment in F0 (*t* test). **b** Percentage of micronucleated cells relative to total cell number in respective generations after treatment with etoposide and controls (ConMN −/ConMN +). The red line indicates the expected rates of micronucleated cells considering the dilution of micronuclei after each mitosis. **c** Number of quiescent (until end of sequence) cells after treatment with etoposide and controls (ConMN −/ConMN +) in generations F0–F3. All presented values are mean out of five experiments with standard error. Asterisk represents *p* < 0.05 compared to ConMN−; delta represents *p* < 0.05 to value of the treatment in F0 (*t* test). **d** Number of dead cells after treatment with etoposide and control (ConMN −/ConMN +) in generations F0–F3. All presented values are mean out of five experiments with standard error. Asterisk represents *p* < 0.05 compared to ConMN −; delta represents *p* < 0.05 to value of the treatment in F0 (*t* test). **e** Images of a quiescent cell over a longer period. **f** Images of a dying cell. White arrow indicates micronucleus

In addition to micronuclei, micronucleated cells were also analysed. As expected, etoposide-treated cells went through mitosis less frequently compared to both control groups (Fig. 2a). Non-micronucleated control cells showed the largest number of mitotic cells as a doubling of cell number with each generation was observed until F4. With each cell division, the number of micronucleated cells is expected to be reduced to 50%, if the micronucleus persists in one daughter cell and the other daughter cell does not contain a micronucleus (see the expected rate of micronucleated cells, red line in Fig. 2b). Indeed, when the rate of micronucleated cells was observed, their number decreased from F0 (only cells harbouring a micronucleus were followed; 100% at F0) to F5 in all groups, but the rate of the decrease varied slightly from the highest dose of etoposide, which showed the slowest decline, to micronucleated control cells, in which this decline was strongest (Fig. 2b). In most cases, the number of micronucleated cells decreased down to 0–5% until F5. Only after treatment with 0.5 µg/ml etoposide, an increase was observed from F4 to F5. Comparing the experimentally observed micronucleus numbers with the expected ones (red line in Fig. 2b), we generally found similar decrease rates in micronucleated control cells and 0.5 µg/l etoposide groups, whereas 1 and 2 µg/ml etoposide treatment caused a higher-than-expected rate of micronucleated cells in all generations.

The number of quiescent cells, i.e. cells with no event like mitosis or cell death occurring until the end of the imaging time of 96 h increased significantly from F0 to F3 in each group (Fig. 2c/e). In F1–F3, micronucleated cells (micronucleated control cells and etoposide treatments) showed higher numbers of non-dividing cells than non-micronucleated control cells, but no dose-dependency could be observed. Cells in F4 and F5 can be expected to show no other event like mitosis or cell death, due to the end of imaging after 96 h, which was also observed (data not shown).

Cell death was also analysed only from F0 to F3 (Fig. 2d/f), as generations F4 and F5 could not always be followed for a complete cell cycle due to the end of imaging after 96 h. Cell death was assumed if cells showed increased chromatin density or loss of clear nuclear border (Fig. 2f). There was a predominantly significant increase of cell death in all micronucleated cells (etoposide treatments and micronucleated control cells) compared to non-micronucleated control cells in these generations. Cell death number reached a maximum in F1 and F2, but decreased again slightly in F3. For non-micronucleated control cells, there was a slow increase of cell death rate from F0 to F3, but it was still low compared to the other groups.

**Fig. 3:**
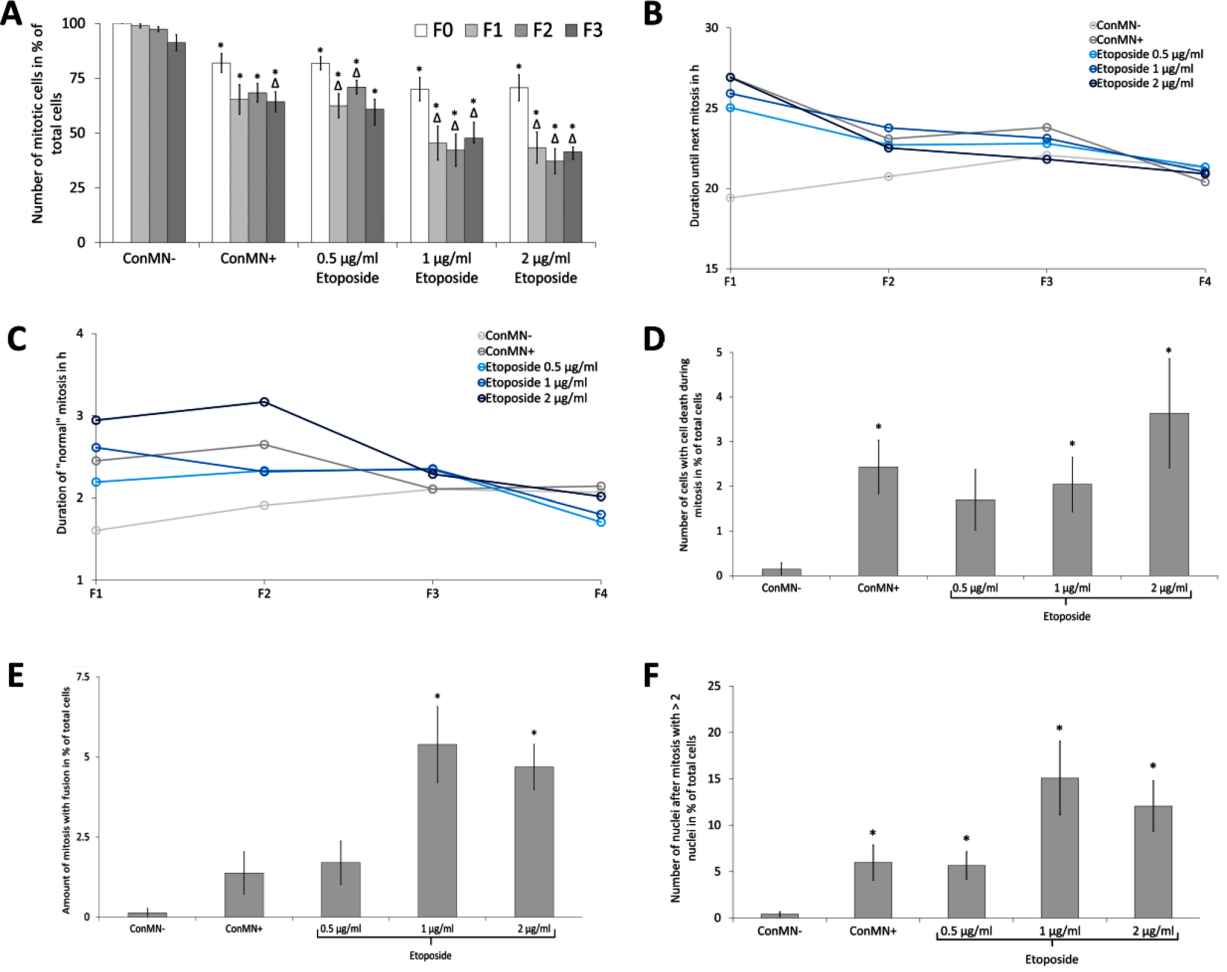
Mitosis and mitotic errors. **a** Number of cells going through mitosis without any mitotic errors during a specific generation in % of total cells after treatment with etoposide and control (non-micronucleated control cells (ConMN −)/micronucleated control cells (ConMN +)) in generations F0–F3. All presented values are mean out of five experiments with standard error. Asterisk represents *p* < 0.05 compared to ConMN −; delta represents *p* < 0.05 to value of the treatment in F0 (*t* test). **b** Duration until next mitosis without any mitotic abnormalities. **c** Duration of this event in generationa F1–F4. **d** Number of mitotic cells with cell death during mitosis in % of total cells. All presented values are mean out of five experiments with standard error. Asterisk represents *p* < 0.05 compared to ConMN − (*t* test). **e** Number of mitosis after fusion of two cells. All presented values are mean out of five experiments with standard error. Asterisk represents *p* < 0.05 compared to ConMN − (*t* test). **f** Number of mitoses resulting in > 2 nuclear fragments after mitosis in % of total cells. All presented values are mean out of five experiments with standard error. Asterisk represents *p* < 0.05 compared to ConMN − (*t* test)

The frequency of mitoses without any errors was evaluated from F0 to F3 (Fig. 3a, Supplementary Fig. 4a). Again, generations F4 and F5 were not considered due to the proximity to the end of imaging. Most non-micronucleated control cells went through mitosis. Although this rate slightly declined with each generation, it was still higher compared to micronucleated cells (micronucleated control cells and etoposide treatments). 1 und 2 µg/ml etoposide treatments caused the lowest numbers of mitoses. The mitosis number decreased significantly further in F1–F3 compared to F0 after all etoposide treatments, but only slightly in micronucleated control cells. The duration of interphase was assessed for all groups from F1 to F4 (Fig. 3b). Whereas interphase in F1 in non-micronucleated control cells lasted only around 20 h, it was 25–27 h in the other groups. Interestingly, this difference became smaller with each generation and in F4 only minor changes between all treatments and controls occurred, when the mean interphase duration was around 21 h. The duration of the process of mitosis itself (from prophase to telophase) showed a similar trend (Fig. 3c): The largest difference was found in F1 with non-micronucleated control cells showing the shortest mitosis (~ 1.5 h) and 2 µg/ml etoposide treatment causing the longest mitosis (~ 3 h). In contrast, only slight differences were found in F4, in which a mitosis duration of ~ 2 h was observed in all groups.

In addition to characterizing normal mitoses, some types of mitotic errors were analysed, specifically cell death during mitosis (Supplementary Fig. 4b), mitoses after fusion of two nuclei, and mitoses resulting in more than two nuclei (Supplementary Fig. 4c). As these events occurred only rarely, a separate evaluation for the different generations is not meaningful and only the sum of these events from all generations was analysed. All these events were very rare in non-micronucleated control cells (0.1–0.4% of all cells), but more frequent in micronucleated control cells and dose-dependently increased after etoposide treatment (Fig. 3d–f). Mitosis with cell death was significantly higher in micronucleated control cells and after 1 and 2 µg/ml etoposide treatments compared to non-micronucleated controls. Up to 3.6% of all cells underwent mitosis with cell death in the 2 µg/ml group. Mitosis after fusion of two nuclei was more frequent, particularly after etoposide treatment (5.4% and 4.7% in the 1 and 2 µg/ml groups). Mitosis with more than two daughter nuclei could be observed in 15% and 12% of all cells after 1 and 2 µg/ml etoposide treatment.

## Discussion

The fate of micronuclei and micronucleated cells is a very important subject, because it addresses the question of the relevance of chromosomal mutations for the organism, which is essential for many biological processes including carcinogenesis. Nevertheless, there is only a small number of studies available explicitly focussing on the fate of micronuclei and micronucleated cells (Hintzsche et al. 2017). The aim of this study was to analyse the consequences of chromosomal DNA damage using live cell imaging for a period of four to five cell divisions, which to our knowledge is longer than all previous studies addressing this question. The most important fates for micronuclei were persistence and, to a smaller extent, reincorporation during the next mitosis. Extrusion and degradation were observed only rarely. Persistence and reincorporation of micronuclei were also described in a number of studies, e.g. a recent study using a photo-switchable fluorescent protein fused to H2B showed persistence of micronuclei in ~ 75% and reincorporation in ~ 25% of all investigated cells one mitosis after micronucleus formation (Soto et al. 2018). Another live imaging study suggested that 16% of all micronucleated cells divided to daughter cells without any micronuclei (Crasta et al. 2012). With HeLa-H2B cells from the same cell line as used in our experiments, 33.8% of the daughter cells of micronucleated cells showed no presence of micronuclei, indicating reincorporation, whereas 50.7% of the daughter cells comprised at least one micronucleus (Utani et al. 2010). After irradiation, it was found that micronucleated cells produced micronucleated daughter cells at a much higher frequency than non-micronucleated cells depending on the cell line (63.1–87.7%), indicating that a certain number of micronuclei persist and a smaller number reincorporate into the main nuclei (Huang et al. 2011b). Many other studies are available showing persistence at least during interphase, suggesting that micronuclei are quite stable until mitosis (Hintzsche et al. 2017).

Degradation of micronuclei was proposed or described by some authors. For example, γH2AX foci inside micronuclei were interpreted as signs of degradation of the chromatin (Terradas et al. 2009). Also, sequestration of Rad51-foci into micronuclei was proposed to demonstrate a way to eliminate damaged DNA from the nuclei, even though a proof of degradation of micronuclei with damaged DNA could not be shown (Haaf et al. 1999). In addition, micronuclei were also shown to be co-localized with markers for autophagy like LC3, which could represent a mechanism of degradation of micronuclei (Rello-Varona et al. 2012). Another study also found a co-localization of LAMP1, a marker for lysosomes, with micronculei (Sagona et al. 2014). Even though these studies detected associations between potential degradation markers and micronuclei, no study could find any convincing evidence for degradation of whole micronuclei in living cells so far. This is in line with our results, showing degradation only rarely or never, which points to a need for further studies, if the association of degradation markers really induces degradation of micronuclei.

Furthermore, extrusion of micronuclei was not or only rarely observed in our experiments either, but more frequently in other studies. Probably, the findings of extrusion of micronuclei can be attributed to unusual experimental or biological circumstances and cannot be generalized. For example, high doses of cytochalasin B lead to extrusion of main nuclei, but also of micronuclei after treatment with an additional genotoxic agent (Nito et al. 1988). Double minutes, small extrachromosomal fragments, were extruded from the cell by low doses of hydroxyurea, which might pose a mechanism for the cell to eliminate unnecessary DNA (Shimizu et al. 2000). Extrusion was also found after treatment with genotoxic substances in mouse polychromatic erythrocytes, mainly after treatment with aneugenic substances (Parton et al. 1991; Schriever-Schwemmer et al. 1997). It is likely that this does not represent a micronucleus-specific mechanism, but rather can be taken as a by-product of the regular (main) nucleus extrusion during erythrocyte maturation. Again, no evidence for extrusion of micronuclei was found in living cells (Crasta et al. 2012; Huang et al. 2012). All in all, extrusion of micronuclei is presumably limited to special conditions and seems generally not to occur in most cell systems.

Although the rate may vary between different cell lines and treatment options, there seems to be a general trend for the majority of micronuclei to persist and, to a smaller extent, to reincorporate into the main nucleus, while degradation and extrusion are not relevant or occur only under special conditions. These findings have a number of consequences for the question whether micronuclei are cause or consequence of carcinogenesis. First, it seems clear that the DNA in micronucleated cells is not lost or removed, which was proposed already several years ago (Huang et al. 2012). Second, it may point to an important role of micronuclei for chromothripsis. Although other explanations exist for why cells undergo chromothripsis (e.g. telomere dysfunction via breakage-fusion-breakage cycles), micronuclei are part of the most likely models explaining chromothripsis (Luijten et al. 2018). It occurs when chromosomes in asynchronous micronuclei are pulverized due to massive DNA damage and the resulting fragments are ligated in random order. During the next mitosis, the ligated chromosome can be reincorporated to the main nucleus and thus introduce multiple genomic alterations within a short time (Crasta et al. 2012). Asynchronous micronuclei might emerge due to disruption of the micronuclear envelope causing an impairment of DNA damage repair and delayed replication (Terradas et al. 2016). The pulverized chromosomes are repaired by non-homologous end joining, which leads to enormous alterations on one or few chromosome(s) or chromosomal regions (Ly et al. 2017). Although the relevance of chromothripsis for carcinogenesis is under discussion, it may demonstrate that reincorporating micronuclei could be an active driver of carcinogenesis (Orr and Maiato 2019). Furthermore, it is still unknown if chromothripsis-like processes also occur after reincorporation of chromosomal fragments. It is very theoretical, if fragments can sustain in the nucleus or if they were degraded during the following cell cycles.

On the other side, the viability of the cell is crucial for the assessment of micronuclei. A lot of parameters were evaluated to judge, if and how micronucleus induction affects cell fate. Frequency of mitosis, cell death, and persistence or abnormal mitosis were altered compared to non-micronucleated cells. Also, the total number of cells per generation, indicating the total growth in a group, is reduced, which became most significant from F2 to F4. While non-micronucleated control cells showed almost a doubling until F4, this rate was decreased in micronucleated control cells beginning in F2 and even more after etoposide treatments, leading to stagnation or reduction of cell number from F3 to F4. Persistence without any further division in F0–F3 can be taken as an evidence for cell arrest, as usually four to five mitoses are expected in HeLa cells after 96 h. As the rate of quiescent cells is increased in micronucleated cells from F0 to F3 regardless of treatment, also cell arrest seems to be a possible fate for micronucleated cells. Besides cell arrest, the cell death rate is increased in micronucleated cells in a dose-dependent manner. There is some evidence that micronuclei could trigger apoptosis, although it is difficult to distinguish whether micronuclei cause apoptosis or if heavily damaged cells tend to form micronuclei as a consequence of general cytotoxicity (Decordier et al. 2002; Utani et al. 2010). Mechanistic investigations revealed that caspase-3 inhibition causes a decrease in micronuclei number, whereas caspase-8 and caspase-9 inhibition increased the amount of micronuclei, which could suggest a role of caspase-3 in micronucleus induction (Decordier et al. 2005). A decrease of apoptosis could not be associated with an increase in micronuclei induction, suggesting a more complicated relationship between apoptosis and micronuclei frequency than expected (Vukicevic et al. 2004).

Not surprisingly, the frequency of mitosis is significantly lower in micronucleated cells and still lower after high dose etoposide treatment. Interestingly, the mitosis rates in these groups remained almost constant from F1 to F3 after a decrease to F0. Regarding the duration of interphase and mitosis, an increase for both phases in micronucleated cells is obvious in F1 and F2, but vanishes in F3, indicating a potential recovery of delayed interphase and mitosis after several cell cycles. This finding is consistent with other studies using the same cell line after treatment with hydroxyurea (Utani et al. 2010). A recent live imaging study in another HeLa cell line also found an increase in mitosis duration in micronucleated cell, specifically cells with kinetochore-positive micronuclei showed a stronger increase than cells with micronuclei without kinetochore (Jiang et al. 2019). Mitotic errors occurred nearly exclusively in micronucleated cells and were furthermore increased after etoposide treatment.

Altogether, these data show that the cell cycle of micronucleated cells is delayed and that the risk for cell death or cell arrest is increased. Nevertheless, micronucleated cells can divide multiple times even after exposure to strong genotoxic agents. Most of the micronuclei persist for one or more cell cycles or are reincorporated during mitosis. Degradation and extrusion of micronuclei are very rare events and it is unclear whether these processes play relevant roles. It is well known that the micronucleus frequency is associated with cancer risk in humans, but, e.g. chromothripsis (and probably also other ways for genome reorganization) may also play an important role in species evolution, when occurring in germline cells (Bonassi et al. 2007; Pellestor and Gatinois 2019).

## Electronic supplementary material

Below is the link to the electronic supplementary material.

Supplementary file1 (DOCX 8525 kb)

## Data Availability

The datasets generated during and/or analysed during the current study are available from the corresponding author on reasonable request.
