# Supplementary material for: Long-term fate of etoposide-induced micronuclei and micronucleated cells in Hela-H2B-GFP cells
Source: Arch Toxicol. 2020 Jul 17;94(10):3553–61. doi: 10.1007/s00204-020-02840-0 (PMC7502055; doi:10.1007/s00204-020-02840-0)
Supplement: Supplementary file 1 — Supplementary file1 (DOCX 8525 kb) [file 204_2020_2840_MOESM1_ESM.docx]

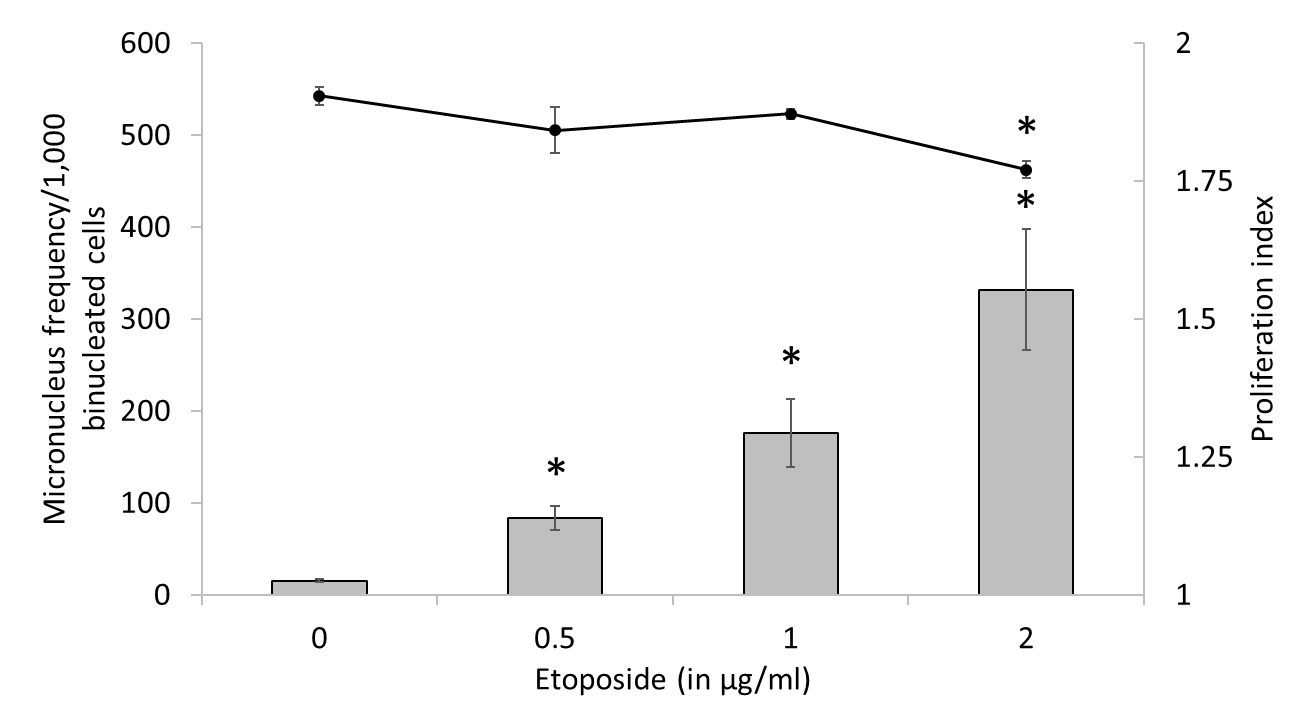


**Supplementary Fig. 1.** Micronucleus frequency (grey bar) per 1,000 binucleated cells and proliferation index (black line) after treatment with etoposide in HeLa H2B-GFP. Mean of four independent experiments ± standard error. Asterisk represent p<0.05 to treatment with 0 µg/mL etoposide (t-test).


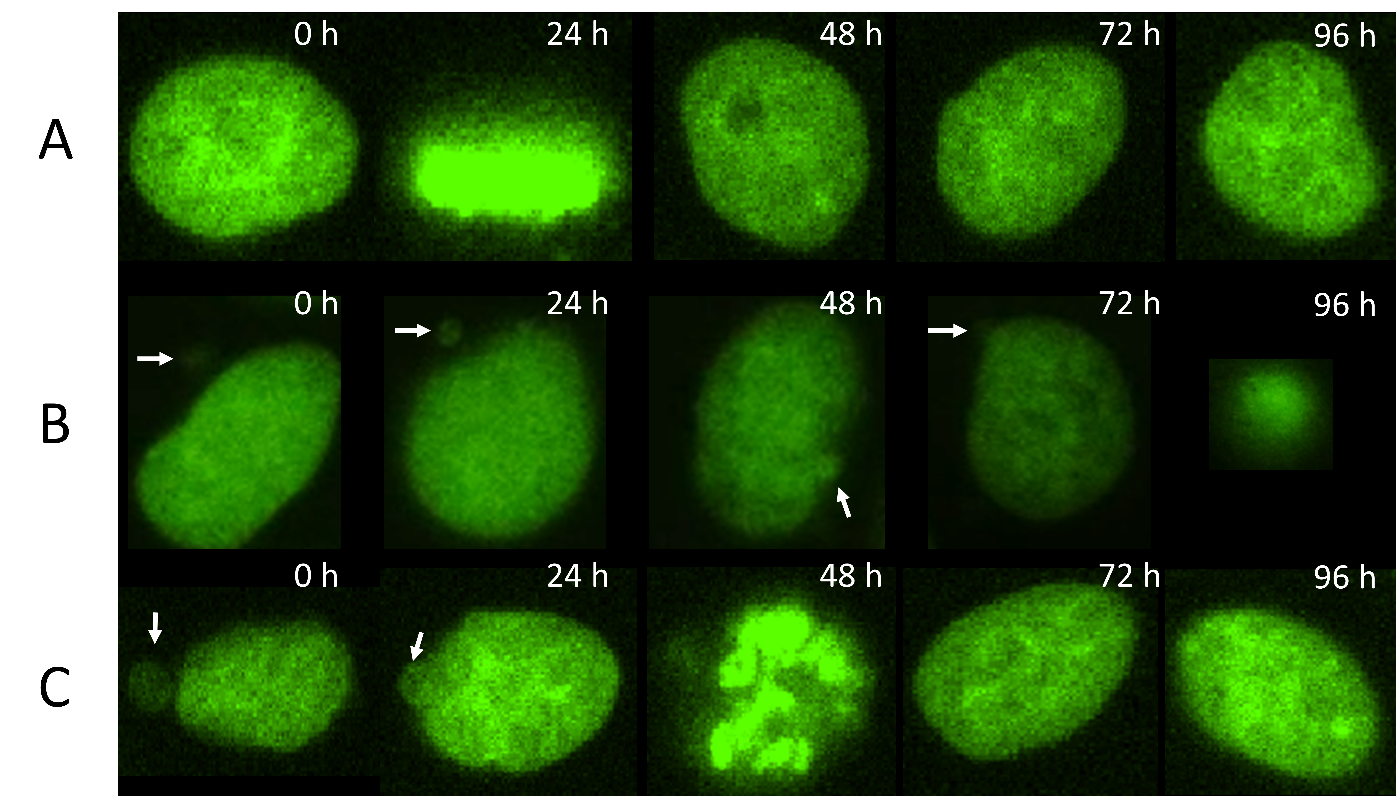


**Supplementary Fig. 2.** Sequences of cell nuclei over the full observation period of 96 hours. White arrows indicate micronuclei. A) Non-micronucleated cell and daughter cells at several time points during the following cell cycles (picture at 24 h shows mitosis). B) Micronucleated cell and daughter cells with persistent micronuclei at several time points during the following cell cycles and cell death occurring just before the end of the observation period. C) Micronucleated cell which formed non-micronucleated daughter cells (one of which is shown) undergoing further cell cycles without cell death (picture at 48 h shows mitosis).





**Supplementary Fig. 3.** Representative sample scheme of a micronucleated cell with subsequent cells with and without micronuclei.


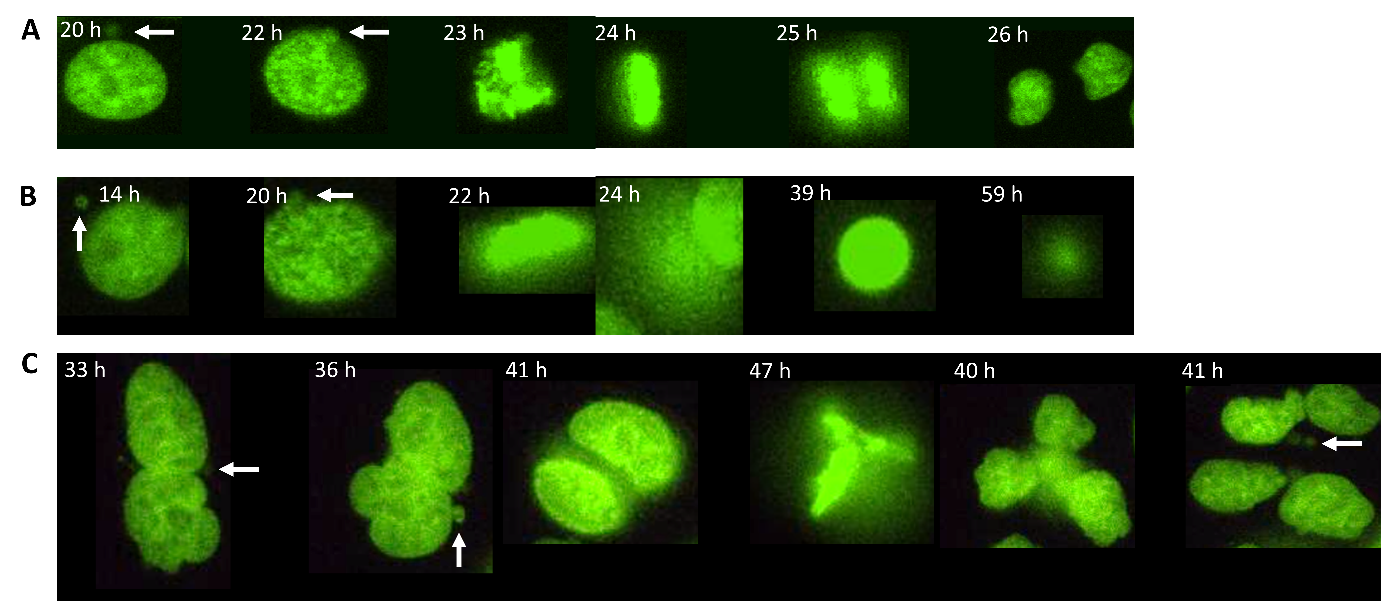


**Supplementary Fig. 4.** Images of events evaluated in this study. White arrows indicate micronuclei. (A) Images of mitosis without any observable mitotic abnormalities. (B) Images of a cell undergoing mitosis with cell death. (C) Images of mitosis after fusion of two nuclei with subsequent division into 4 nuclei.
